# A technical review of percutaneous sclerotherapy with bleomycin for giant hepatic venous malformation

**DOI:** 10.1186/s42155-023-00394-7

**Published:** 2023-09-27

**Authors:** Omid Ghaemi, Mohammad-Mehdi Mehrabi Nejad, Mohammad Reza Rouhezamin, Niloofar Ayoobi Yazdi, Ramin Pourghorban, Hadi Rokni Yazdi

**Affiliations:** 1 Department of Radiology, Advanced Diagnostic and Interventional Radiology Research Center (ADIR), Medical Imaging Center, Imam Khomeini Hospital, Tehran University of Medical Sciences (TUMS), Qarib St, Keshavarz Blvd, Tehran, 1419733141 Iran; 2 Interventional Radiologist, Tabesh Imaging Center, Shiraz, Iran; 3 Department of Radiology, Sina Hospital, Tehran University of Medical Sciences (TUMS), Tehran, Iran; 4 Department of Medical Imaging, Nepean Hospital, Kingswood, NSW Australia

**Keywords:** Liver, Vascular malformation, Haemangioma, Sclerotherapy, Bleomycin

## Abstract

**Background:**

Hepatic venous malformation (HVM), traditionally called liver haemangioma, is considered the most common benign hepatic lesion. Treatment might be indicated in large and symptomatic HVMs. We aim to describe stepwise technical aspects of trans-hepatic percutaneous sclerotherapy of hepatic venous malformation (HVM).

**Main text:**

Patients with symptomatic HVM larger than 5 cm are selected after discussion in hepatobiliary multidisciplinary team. After prophylactic antibiotic and corticosteroid administration, local anaesthesia and conscious sedation are applied. A 22-gauge spinal or Chiba needle is used to obtain percutaneous access to the HVM through normal liver parenchyma under ultrasound guidance. To ensure proper needle placement and to prevent accidental delivery of sclerosant into unintended areas, about 5–10 mL iodine contrast is injected under fluoroscopy. Then, 45–60 IU bleomycin is mixed with 10 mL distilled water and 10 mL lipiodol and is slowly injected under fluoroscopy over a period of 20–30 s. After the needle is removed, manual pressure is applied over the puncture site for a period of 5 min followed by placement of a sandbag. Patients are monitored for 6–8 h post-procedure.

**Conclusion:**

In this technical review, we described our institutional technique of percutaneous sclerotherapy, which could be regarded as an alternative to TAE in the management of HVM.

## Background

Hepatic venous malformation (HVM), traditionally called liver haemangioma, is considered the most common benign hepatic lesion [1]. HVMs are usually small and stable, and do not require active management. However, treatment might be indicated in large and symptomatic HVMs [2]. Surgical resection, trans-catheter arterial embolization (TAE), radiofrequency or microwave ablation, and percutaneous sclerotherapy are different treatment options [2]. Danza et al. [3] firstly reported the use of percutaneous sclerotherapy with ethanol for the treatment of HVMs in two patients. Thereafter, Rokni Yazdi et al. [4–6] further developed this technique, reporting positive results with reduction in the size and improvement in symptoms with no major complication.

Various sclerosing agents are used in different methods, including lipiodol and bleomycin, Pingyangmycin (bleomycin A5 hydrochloride), or ethanol, which are also commonly utilised agents in TAE and percutaneous sclerotherapy [7]. Bleomycin, an mTOR inhibitor, has been used as a treatment for vascular malformations like HVMs [8]. Lipiodol, which has drug delivery and radio-opacity characteristics, is widely used in TAE, but its vital role in percutaneous sclerotherapy remains unclear [7].

The aim of this pictorial and technical review is to describe the stepwise technical aspects of trans-hepatic percutaneous sclerotherapy of HVM.

## Main text

### Patient selection

The diagnosis of HVM is made based on characteristic imaging findings, including centripetal peripheral nodular enhancements on triphasic abdominal computed tomography (CT) scans. HVM with at least one diameter greater than 5 cm is considered giant HVM. Symptomatic patients are selected for this procedure after discussion in a multidisciplinary meeting, while those who exhibit hepatic or renal impairment, other potential causes of symptoms, pre-existing pulmonary fibrosis, or allergy to contrast media are excluded. The international normalized ratio > 1.5, platelet count < 50,000/µL, or partial thromboplastin time > 1.5 times of control are corrected before proceeding with the procedure. We are a referral hospital that has successfully treated over 300 patients with HVM, averaging 2 patients per week.

### Pre-procedural considerations

This single-session procedure is performed in an outpatient setting under local anaesthesia and conscious sedation in our institute. The patient’s vital signs and cardiac rhythm are continuously monitored during the procedure. After sterilising the skin, about 10 mL local anaesthetic (lidocaine hydrochloride 2%) is administrated around the capsule, intercostal muscles, and the overlying subcutaneous tissue. For conscious sedation, midazolam (1 mg) and fentanyl (100 µg) are used for induction followed by a combination of ketamine (5 mg) and propofol (10 mg) for the maintenance. To reduce the risk of infection and delayed hypersensitivity reaction to bleomycin, prophylactic antibiotic (1 gr of cefazolin, intravenous) [9] along with a single dose of corticosteroid (1 vial of Depo-medrol 40 mg/ml, intramuscular) are administered, respectively, 30 min before the procedure.

### Percutaneous puncture

**Fig. 1:**
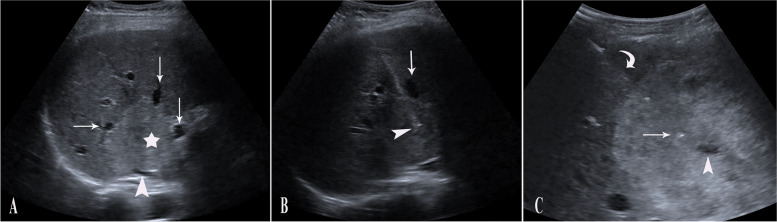
** A** Transverse liver ultrasound scan demonstrating a hepatic venous malformation (HVM) (marked by a star) that is compressing the inferior vena cava (arrowhead) and hepatic veins (arrows). **B** A 22-gauge Chiba needle (arrowhead) is passing between hepatic veins (red arrow) into the haemangioma, with a significant portion of normal liver tissue transgressed to reduce the risk of bleeding. **C** Another patient with HVM during ultrasound-guided access, in which the intervening normal liver parenchyma is notable (curved arrow), and the needle tip (red arrow) is slightly away from the cystic area (arrowhead)

The first step involves ultrasound-guided puncture of the HVM, as illustrated in Fig. 1. To obtain percutaneous access to the HVM, a fine spinal or Chiba needle with a gauge of 22 is preferred. To minimize the risk of bleeding, the needle is passed through normal liver parenchyma before entering the HVM. In rare instances when normal parenchyma is not detectable between the mass and the liver capsule, other treatment options including surgery or trans-arterial embolization might be preferred.

**Fig. 2:**
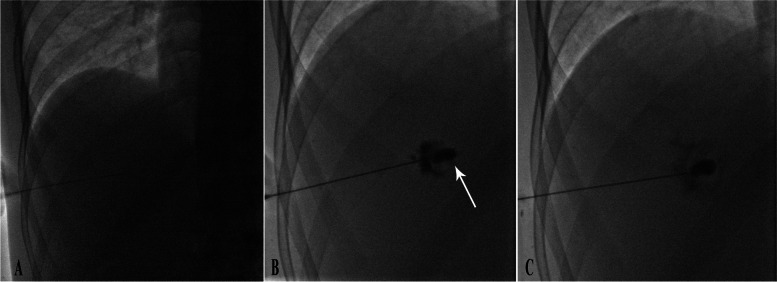
** A** The lesion is punctured under ultrasound and fluoroscopy guidance. **B** Contrast injection shows the needle tip in a central cystic area (arrow). Note that the contrast remains in this area and does not spread inside the haemangioma. **C** The needle is slightly withdrawn, now showing further dilution and dissemination of the contrast agent

**Fig. 3:**
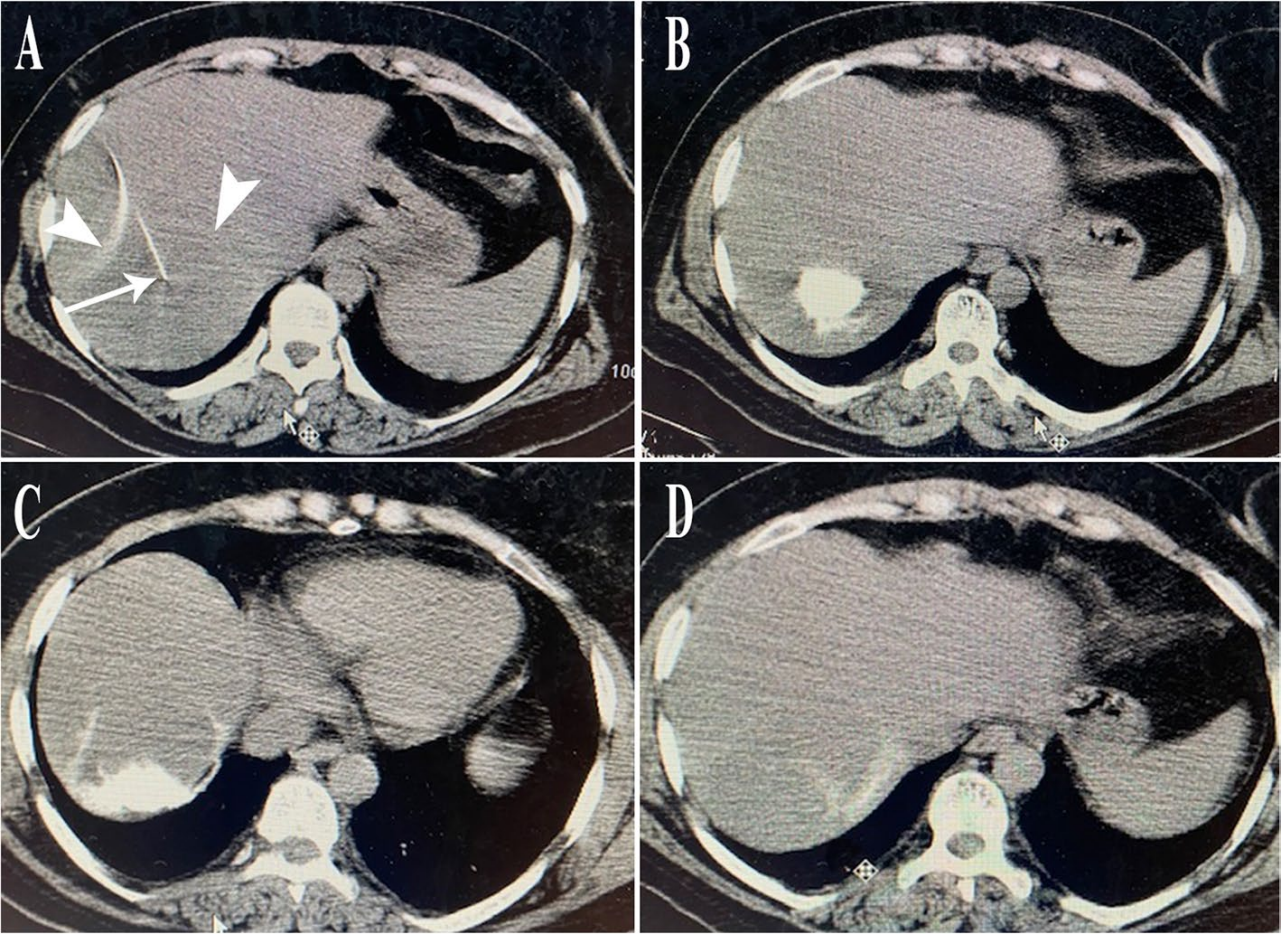
** A** Hepatic venous malformation (arrowheads) in the posterior right lobe of a middle-aged woman is accessed with a 22-gauge Chiba needle under CT guidance. The tip of the needle is marked with an arrow. **B** Water-soluble contrast is injected into the lesion. **C**, **D** Slow infusion of bleomycin solution shows the dissemination of contrast/bleomycin mixture within the lesion after 2 and 4 min, respectively

The optimal target for HVM treatment is the centre of the lesion, avoiding areas of scar or calcification. Central scarring does not appear to have a significant connection with the venous channels of the HVM (Fig. 2). If pressure effect is detected on adjacent vascular or non-vascular structures, targeting the HVM near the compressed structure is preferred as a more effective response in this location is desired. Ultrasound guidance is the preferred imaging modality in our centre, as it enables real-time navigation of the needle without the use of ionizing radiation. However, CT guidance may be used if a safe route is not identifiable on ultrasound. Some interventionists may opt for CT or CT fluoroscopy as the primary guidance modality (Fig. 3).

**Fig. 4:**
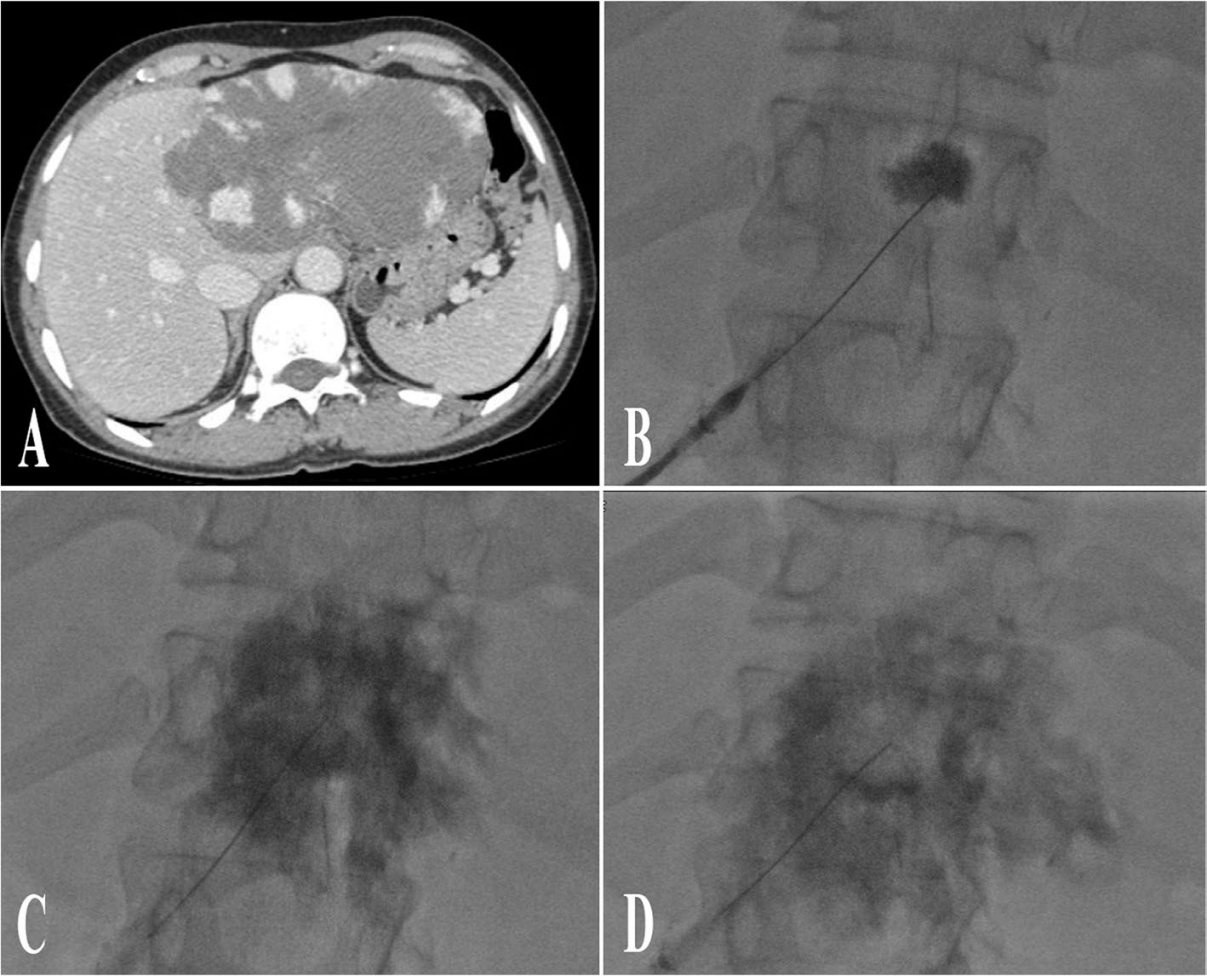
** A** Giant HVM with typical enhancement in the left liver lobe in a middle-aged woman complaining of chronic abdominal pain. **B** After ultrasound-guided access, contrast injection with fluoroscopic surveillance is performed to check the needle tip position. **C**, **D** Injection of bleomycin solution shows gradual rarefaction and dissemination of contrast

To confirm the needle tip position and to prevent accidental delivery of sclerosing agent into the biliary tract or vascular structures, approximately 5–10 mL water-soluble iodine contrast is injected under real-time fluoroscopy. A typical hallmark of HVM is slow centrifugal spread of the contrast agent with a speculated border, as seen in Fig. 4. In rare cases, delayed small venous channel filling may be observed. If early opacification of the bile ducts, portal vein, or hepatic vein is observed, the needle tip is repositioned to avoid possible complications.

### Sclerotherapy

Bleomycin is a commonly used sclerosant for treating HVMs through percutaneous injection. The standard dose of bleomycin used in our centre for a single-session treatment ranges from 45 IU to 60 IU (Bleocin-S; Korea United Pharm Inc., South Korea). The sclerosing agent is diluted with 10 mL distilled water and then is mixed with 10 mL lipiodol or only diluted with 20 mL distilled water without lipiodol using a 20 mL syringe with a three-way stopcock. The prepared bleomycin-lipiodol mixture is then slowly injected under fluoroscopy over a period of 20–30 s.

Sclerosing agents vary in different methods and centers. There is no general consensus on the best sclerosing agent to be used. However, the traditional usage of polyvinyl alcohol (PVA) showed disappointing results in reducing patients’ symptoms and HVM size (< 1 cm in the largest diameter), lipiodol-based treatments with bleomycin, pingyangmycin or ethanol showed several favorable outcomes [7]. Pingyangmycin is not approved by FDA in western countries, so it is barely available in some regions and ethanol is not preferred due to its potential toxicity [10]. Therefore, bleomycin, which was previously confirmed for treatment of vascular malformations, is widely recommended [11]. In a recent meta-analysis on 21 studies, lipiodol-based treatments showed some significantly better outcomes in decreasing the HVMs size compared to PVA embolization [7].

Lipiodol is radio-opaque and is helpful to initiate, improve, and optimize the therapeutic process as a drug delivery system [12]. Moreover, it is radio-opaque, so it can provide interventionists with a better vision in terms of fluoroscopy guidance. However, Lipiodol proves to be rather costly, prompting us to adapt our technique and solely utilize bleomycin. As mentioned earlier in the sclerotherapy technique, after accessing the haemangioma, 5–10 mL contrast agent is injected to evaluate potential connections. Therefore, the injected bleomycin-distilled water solution displaces the previously injected contrast agent from the central part of the HVM towards its peripheral parts, eventually fading away. This allows the interventionist to track the injection of bleomycin as a negative contrast. So, currently we do not use lipiodol. Besides, multiple punctures are avoided as it may pose an increased risk of bleeding.

### Post-procedural consideration

After the needle is removed, manual pressure is applied to the site of puncture for a period of 5 min. Subsequently, a sandbag is placed over the puncture site, and the patient is transferred to the recovery room [13, 14].

Patient is monitored for a duration of 6–8 h post-procedure to identify any relevant new signs or symptoms, as well as periprocedural complications. The procedure is deemed technically successful following the administration of the sclerosing agent into the HVM, after ensuring that there is no connection with either the vascular structures or the biliary systems. In addition, an abdominal ultrasonography is performed to evaluate the presence of new intra-abdominal free fluid or haematoma around the puncture site before discharge. Patient is advised to report any related symptoms to the department.

### Follow-up

**Fig. 5:**
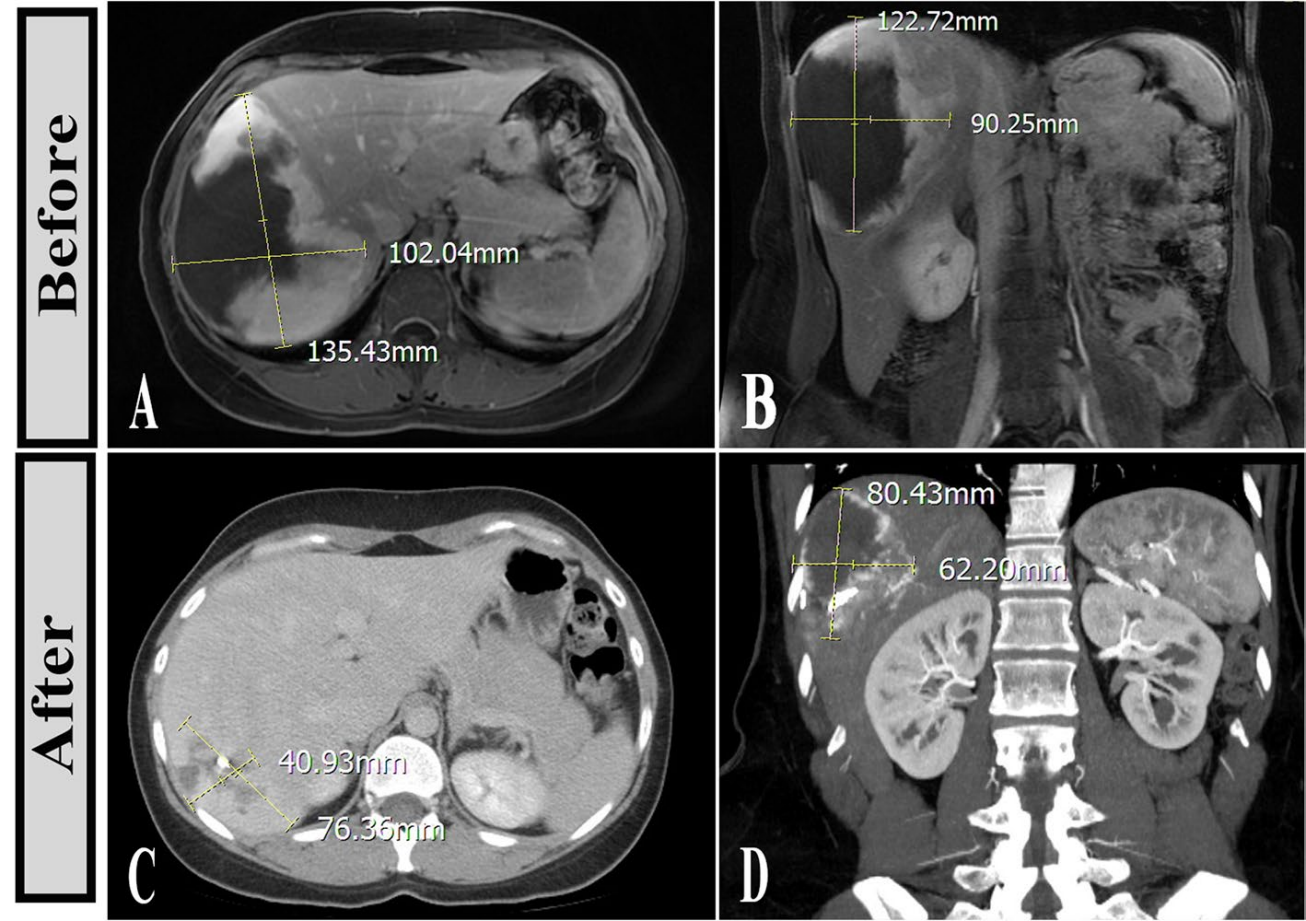
** A**, **B** MRI of a 37-year-old woman with vague abdominal pain, showing a giant HVM in the right liver lobe with typical enhancement. **C**, **D** Nine months after treatment with bleomycin and lipiodol, significant reduction in size is notable. Small clumps of lipiodol are seen within the lesion on control CT scan as dense spots

**Fig. 6:**
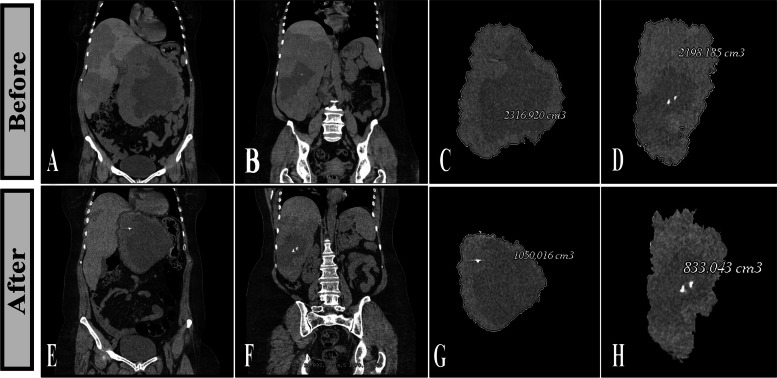
** A, B** A middle-aged woman with two giant HVMs in the left and right liver lobes with abdominal pain and early satiety. **C**, **D **Volumetry of the left and right lobe HVMs depicts HVM volume of 2316 mL and 2198 mL before the procedure, respectively. **E**, **F** Follow-up CT nine months after sclerotherapy shows size reduction in both lesions. **G**, **H** Post-treatment volumetry shows more than 50% volume loss in both lesions, with a volume of 1050 and 833 mL in the left and right lesions, respectively. The patient reported significant relief of upper abdominal pain

Complete blood count, coagulation tests, and liver function tests were measured after 24 h of the procedure as well as at 6- and 12-month follow-ups. The optimal clinical and imaging outcomes are anticipated to take place approximately 6 months after the procedure. Consequently, a 6-month follow up CT or MRI is performed. Patient who undergoes treatment with lipiodol is expected to exhibit high-density material on CT scan and low-signal areas on MRI at the injection site. Anticipated interval changes include a reduction in lesion size, as well as symptoms relief and improvement of pressure effect on adjacent organs (Figs. 5 and 6). During the initial follow-up visit after 6 months, if the patients’ symptoms improved, it is deemed to have a successful clinical outcome. If the symptoms persist, a second session with the same protocol may be arranged after consultation with the multidisciplinary tumour board.

### Complications

The majority of procedure-related complications are minor and do not necessitate alterations to the treatment plan or a prolonged hospital stay. After the procedure, patient may experience mild pain at the puncture site and short-term radiating pain in the right shoulder. Some patients may experience mild and vague pain and heaviness in the upper abdomen for 4–5 days post-procedure, which can be managed conservatively. However, if the pain persists or intensifies, a thorough imaging evaluation is recommended. Additionally, post procedural nausea and vomiting are managed with watchful waiting and intravenous administration of antiemetic drugs, such as Ondansetron 4 mg.

**Fig. 7:**
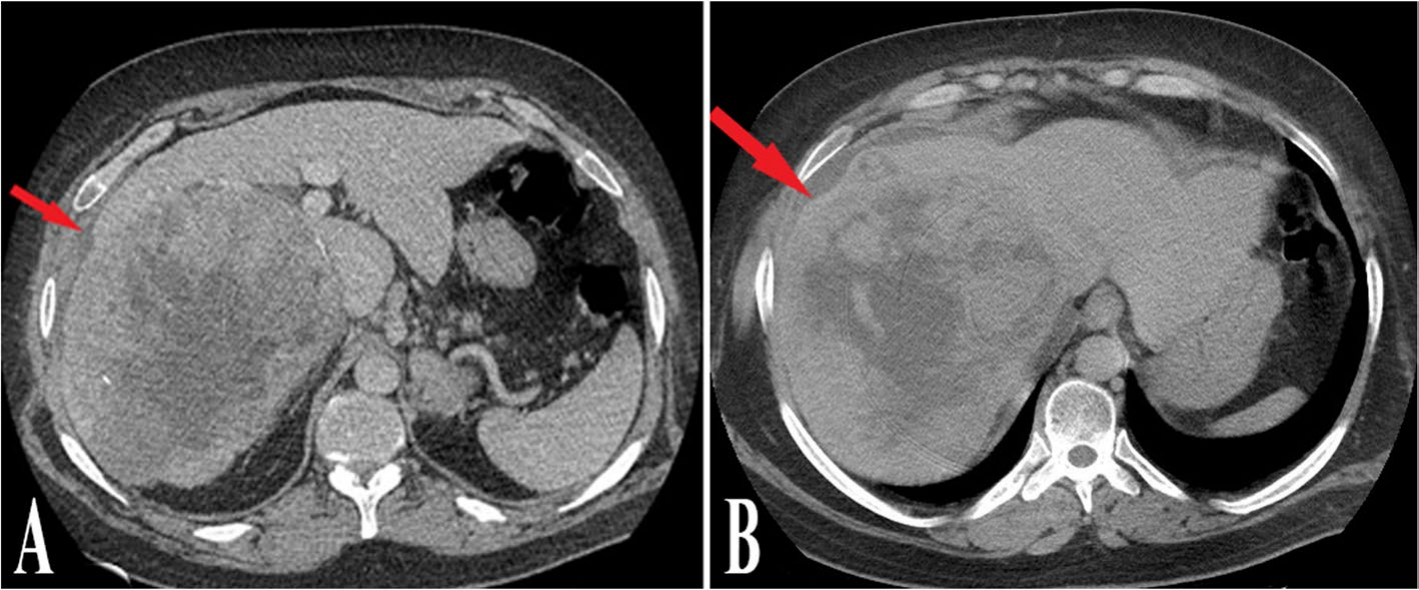
** A, B** Small subcapsular hematoma (red arrows) after puncture of HVM with a Chiba needle, which resolved spontaneously. The patient was monitored for 24 h with no major complication before discharge. Passing a thin rim of liver parenchyma was likely the cause of the haemorrhage

Rare cases of bleeding after the percutaneous needle insertion have been documented, often resulting from direct puncture of the HVM without passing through normal liver parenchyma and/or insufficient pressure application during the early post-procedural period (Fig. 7). In such cases, careful assessment of vital signs, urgent imaging evaluation, and hospital admission are necessary. Our 6-year experience with this procedure has indicated no instances of mortality or major morbidity.

## Conclusion

In this technical review, we described our institutional technique of percutaneous sclerotherapy, which could be regarded as an alternative to TAE in the management of HVM.

## Data Availability

Not applicable.

## Abbreviations

HVM: Hepatic venous malformation
TAE: Trans-catheter arterial embolization
CT: Computed tomography
